# Meta-analysis of the effect of nafamostat mesilate versus no-anticoagulation regimens in adult patients undergoing continuous renal replacement therapy

**DOI:** 10.3389/fmed.2026.1984576

**Published:** 2026-10-21

**Authors:** Yao Wang, Xiaoli Du, Dan Wen, Rong Xu, Xiao Yu, Lingning Zhao

**Affiliations:** Intensive Care Unit, School of Medicine, Mianyang Central Hospital, University of Electronic Science and Technology of China, Mianyang, Sichuan, China

**Keywords:** acute kidney injury, anticoagulant, continuous renal replacement therapy, ICU, nafamostat mesilate

## Abstract

**Background:**

Continuous renal replacement therapy (CRRT) is a critical treatment for critically ill patients with acute kidney injury. However, effective anticoagulation of the extracorporeal circuit remains a clinical challenge. Nafamostat mesilate (NM), a serine protease inhibitor, possesses localized anticoagulation properties; however, its efficacy and safety in high-bleeding-risk populations have not been systematically evaluated.

**Objective:**

To compare the efficacy and safety of NM anticoagulation and no-anticoagulation regimens in patients undergoing CRRT.

**Methods:**

Randomized controlled trials (RCTs) and cohort studies were retrieved from PubMed, Web of Science, MEDLINE, Cumulative Index to Nursing and Allied Health Literature, the Cochrane Library, Wanfang Database, Chinese National Knowledge Infrastructure, and the Chinese Biomedical Literature Database. We included studies on adult patients receiving CRRT where the intervention and control groups were treated with NM anticoagulation and no-anticoagulation regimens, respectively. Primary outcomes included filter lifespan, number of filters used, coagulation indicators, bleeding events, and in-hospital mortality. The meta-analysis was conducted using RevMan 5.4 software.

**Results:**

Eight studies involving 969 patients were included. The meta-analysis demonstrated that, compared with the control group, the NM group had a significantly prolonged filter lifespan (*P* = 0.01) and a reduced number of filters used (*P* < 0.0001). The activated partial thromboplastin time (*P* = 0.89), prothrombin time (*P* = 0.88), and international normalized ratio (*P* = 0.60) showed no significant differences. Both groups demonstrated clinically similar risks of bleeding events (*P* = 0.53). However, the NM group demonstrated a significantly reduced in-hospital mortality, compared with the control group (*P* = 0.02).

**Conclusion:**

The NM anticoagulation regimen effectively extended the CRRT filter lifespan, reduced resource consumption, and improved patient survival outcomes without increasing the bleeding risk, demonstrating particular value for high-bleeding-risk populations. Future multicenter RCTs are warranted to validate applicability of NM in non-East Asian populations.

**Systematic Review Registration:**

https://www.crd.york.ac.uk/PROSPERO/view/CRD420261399024, identifier: PROSPERO CRD420251077578

## 1 Introduction

Acute kidney injury (AKI) occurs in 5%–70% of critically ill patients, with 14%–30% requiring renal replacement therapy (1). Continuous renal replacement therapy (CRRT) has emerged as a vital intervention for patients with AKI owing to its hemodynamic stability and high-efficiency solute clearance capacity (2). The cornerstone of CRRT implementation depends on effective anticoagulation in the extracorporeal circuit (3). The contact of the blood with artificial surfaces activates the coagulation system, resulting in premature clotting and obstruction of filters and tubing, ultimately leading to interruption of therapy, increased costs, and a heightened nursing burden (4).

Currently, various anticoagulation options are utilized in clinical settings, with unfractionated heparin being the most commonly used anticoagulant; however, it carries significant risks of systemic hemorrhage and thrombocytopenia (5). Regional citrate anticoagulation (RCA) has emerged as the recommended preferred anticoagulation regimen owing to its superior efficacy and lower systemic bleeding risk (6). However, RCA requires vigilant monitoring of ionized calcium levels and the acid-base balance to prevent metabolic complications. Therefore, for patients at a high risk of bleeding, guidelines recommend an anticoagulant-free protocol (7). This strategy involves increasing the saline priming volume and performing scheduled saline flushes; however, a recent RCT reported that frequent saline flushing did not effectively prevent circuit clotting and was associated with increased hypotension and reduced delivered CRRT dose (8). Consequently, anticoagulant-free protocols still significantly shorten the filter lifespan and lead to frequent circuit clotting events (7).

Nafamostat mesilate (NM), a synthetic serine protease inhibitor, demonstrates unique potential as an anticoagulant for CRRT in critically ill patients, and may be especially suitable for those at high risk of bleeding. NM exerts its anticoagulant effect primarily by reversibly and directly inhibiting key enzymes involved in the coagulation cascade (9). Compared with conventional anticoagulants, NM has an ultrashort half-life and undergoes rapid hydrolysis by plasma esterases, which confines its anticoagulant effect primarily to the extracorporeal circuit, thereby enabling highly regionalized anticoagulation with minimal systemic bleeding risk (10). NM is currently predominantly used in Asian regions.

However, existing studies comparing the effects of NM with no-anticoagulation regimens have yielded inconsistent conclusions. Some studies have shown that NM can significantly extend the lifespan of filters and reduce the coagulation rate (11). Some studies suggest that its benefits may be partially offset by potential side effects, such as hyperkalemia and allergic reactions (12). Currently, high-quality systematic reviews that clearly compare the efficacy and safety of NM in high-bleeding-risk populations are lacking. Therefore, this meta-analysis aimed to evaluate the efficacy and safety of NM compared to a no-anticoagulation regimen in adult critically ill patients undergoing CRRT, with a specific focus on interpreting outcomes in the context of patient bleeding risk.

## 2 Materials and methods

The review and analysis were conducted in accordance with the Preferred Reporting Items for Systematic Reviews and Meta-Analyses guidelines (13). The research protocol has been registered in International Prospective Register of Systematic Reviews (registration number: CRD420251077578).

### 2.1 Retrieval strategy

Data from PubMed, Web of Science, MEDLINE, Cumulative Index to Nursing and Allied Health Literature, the Cochrane Library, Wanfang, Chinese National Knowledge Infrastructure, and the Chinese Biomedical Literature Database were retrieved. The retrieval period was from the establishment of the database until May 30, 2025. Specific search strategies were formulated for each database, and the search was conducted by combining subject terms with free terms, as follows:

(“continuous renal replacement therapy” OR CRRT OR “continuous venovenous hemofiltration” OR CVVH OR “continuous venovenous hemodialysis” OR CVVHD OR “continuous hemodiafiltration” OR CHDF OR “renal replacement therapy” OR “Hemofiltration” OR “hemodialysis”) AND (“Nafamostat” OR “Nafamostat Mesilate” OR “anticoagulation”). We also manually searched the references of the reviews and preliminarily included literature. No language restrictions were imposed on the included literature.

### 2.2 Inclusion and exclusion criteria

The following literature inclusion criteria were formulated in accordance with the Population, Intervention, Comparison, and Outcome principle: (1) Study type: Randomized controlled trial (RCT)or cohort study (prospective or retrospective); (2) Participants: adult critically ill patients (aged≥18 years) receiving CRRT; (3) Intervention group: NM anticoagulation ​alone; Control group: no-anticoagulation regimen; (4) Outcome: Reporting at least one core outcome among filter lifespan, number of filters used, coagulation parameters, bleeding events, and in-hospital mortality. We excluded studies involving pediatric patients and studies in which the control group was administered heparin or sodium citrate as the anticoagulant. Studies that were published only as conference abstracts, case reports, or letters to the editor were also excluded.

### 2.3 Literature screening and data extraction

Literature screening was conducted independently by two reviewers (Yao Wang and Dan Wen) according to the predefined inclusion and exclusion criteria. The retrieved records were imported into the reference management software, NoteExpress V4.0 (AegeanSoftware, Beijing, China), to automatically remove duplicates. Subsequent screening was performed based on the titles and abstracts to exclude irrelevant studies.​ The full texts of the studies that passed the initial screening were obtained. These studies underwent secondary assessment using the predefined inclusion and exclusion criteria. Any disagreements between the two reviewers regarding inclusion were resolved by discussion to reach a consensus.​ If disagreements remained unresolved, a third reviewer was consulted for adjudication. Data extraction was performed using standardized forms completed independently by the same two reviewers and then crosschecked. The extracted data included the first author's name, study design, country, research participants, sample size, nafamostat dosage, and outcome measures. We contacted the first or corresponding authors to obtain the relevant information on missing data in the articles. All extracted data were stored in a standardized database (Microsoft Excel 2021).

### 2.4 Risk-of-bias assessment and methodological quality assessment

Two reviewers independently assessed the quality of the included studies using standardized tools. For the RCTs, the Cochrane Risk of Bias tool (version 1.0) was used to evaluate random sequence generation, allocation concealment, blinding of participants and personnel, blinding of outcome assessment, incomplete outcome data, selective reporting, and other biases (14). A three-tiered classification system of low risk of bias–high risk of bias–unclear for item-by-item assessment was adopted. The risk of bias in cohort studies was assessed using the Risk of Bias in Nonrandomized Studies of Interventions (ROBINS-I) tool (15). This instrument evaluates bias across seven domains: confounding, selection of participants, classification of interventions, deviations from intended interventions, missing data, measurement of outcomes, and selection of reported results. Based on the potential for bias in these domains to materially affect the outcomes of interest, cohort studies were categorized as having “low,” “moderate,” “serious,” or “critical” risk of bias. When the two reviewers disagreed on literature quality assessment, the discrepancy was resolved by consultation with a third reviewer. We assessed the potential publication bias of the meta-analysis by using Egger's tests (16).

### 2.5 Statistical analysis

Statistical analyses were performed using the Review Manager 5.4 software provided by the Cochrane Collaboration (Oxford, UK) and STATA statistical software (version 18.0). Continuous variables were expressed as mean difference (MD) or standardized mean difference (SMD) with 95% confidence intervals (CIs). Dichotomous variables were presented as odds ratios (ORs) with 95% CIs. Heterogeneity among the included studies was assessed using the *χ*^2^ test and I^2^ statistic. If *P* > 0.1 and I^2^ < 50%, no significant heterogeneity was observed among the studies, and a ​fixed-effects model​ was applied. If *P* ≤ 0.1 ​or​ I^2^ ≥ 50%, significant heterogeneity existed across studies, and a ​random-effects model​ was adopted. *P* values <0.05 were considered statistically significant. We analyzed the ICU patients and the high-risk bleeding patients as two separate subgroups to address two different clinical scenarios. One is the general intensive care environment where nafamostat is commonly used, and the other is specific groups with strict contraindications to conventional anticoagulation therapy.

## 3 Results

### 3.1 Literature search results

The initial database search yielded 307 potentially relevant studies. After removing duplicates, 195 studies remained for preliminary screening. Through title and abstract review, 32 studies were selected for full-text retrieval and evaluation. Following a full-text assessment, eight studies (17–24) were ultimately included in the meta-analysis: two RCTs (17, 20) and six cohort studies (18, 19, 21–24). The literature selection process is described in detail in Supplementary Material 1.

### 3.2 Characteristics and quality of the included studies

The included studies enrolled 969 patients, with 420 receiving anticoagulation therapy using NM (NM group) and 549 receiving no anticoagulant therapy (NA group). All studies were conducted in South Korea or China and primarily involved critically ill patients receiving CRRT in ICUs. Detailed information on study design, population, CRRT parameters, NM dosage, and reported outcomes is provided in Table 1. Regarding the risk of overall bias in cohort studies, two studies were judged to have low risk, and four studies were judged to have moderate risk (Table 2). The higher risk was usually attributed to confounding, selection of participants, and measurement of outcomes.

**Table 1:** General characteristics of included literature.

Literature	Country	Study Type	Sample Size (NM group/Control)	Population Characteristics	CRRT Mode	NM Maintenance Dose	CRRT Technical Parameters	Primary Outcomes
Hwang et al. (18)	Korea	Retrospective Cohort Study	25 vs 131	Critically ill ICU patients requiring CRRT	CVVH	10-30 mg/h	Filter: AN69; Blood flow rate: 100-150 mL/min; Dialysate flow rate: 1000 mL/h	Filter lifespan, coagulation parameters, bleeding events, survival rate
Lee et al. (20)	Korea	RCT	36 vs 37	CRRT patients with high bleeding risk	CRRT	10–30 mg/h	Filter: AN69; Blood flow rate: 130–200 mL/min; Dialysate flow rate: None	Mortality, transfusion requirements, adverse reactions
Shi et al. (21)	China	Retrospective Cohort Study	36 vs 36	Septic patients with AKI	CRRT	0.4 mg／(kg·h)	/	Coagulation parameters, renal function indicators, number of filters used, bleeding events, hyperkalemia
Zhang et al. (22)	China	Retrospective Cohort Study	14 vs 19	ICU patients requiring CRRT therapy	CVVH	10–35 mg/h	Filter:​ AN69ST; Blood flow rate:​ 150–200 mL/min; Dialysate flow rate: 1000 mL/h	Filter lifespan, coagulation parameters, hospitalization costs
Zhou et al. (23)	China	Retrospective Cohort Study	12 vs 15	Post-cardiovascular surgery CRRT patients with high bleeding risk	CRRT	25 mg/h	/	Mortality, filter lifespan, coagulation parameters
Zhu et al. (24)	China	Retrospective Cohort Study	21 vs 42	Septic patients with AKI and high bleeding risk	CVVH	0.4 mg/（kg·h）	Filter: AN69; Blood flow rate: 150–200 mL/min; Dialysate flow rate: 1000 mL/h	Number of filters used, coagulation parameters, renal function indicators, bleeding events
Choi et al. (17)	Korea	RCT	31 vs 24	AKI patients with high bleeding risk	CRRT	10–30 mg/h	Filter: Ultraflux® AV 600S; Blood flow rate: 150–200 mL/min; Dialysate flow rate: None	Filter lifespan, number of filters used, mortality
Kim et al. (19)	Korea	Retrospective Cohort Study	245 vs 245	Adult patients receiving CKRT	CRRT	10 mg/h	Filter: AN69ST; Blood flow rate: 100–150 mL/min; Dialysate flow rate: 1000 mL/h	Mortality, filter lifespan, ICU length of stay

**Table 2:** ROBINS-I assessment of study bias for cohort studies.

Bias Domain	Hwang et al. (18)	Kim et al. (19)	Shi et al. (21)	Zhang et al. (22)	Zhou et al. (23)	Zhu et al. (24)
Due to confounding	Low	Low	Moderate	Low	Low	Moderate
Selection of participants	Low	Low	Low	Moderate	Moderate	Low
Classification of interventions	Low	Low	Low	Low	Low	Low
Deviation from intended interventions	Low	Low	Low	Low	Low	Low
Missing data	Low	Low	Low	Low	Low	Low
Measurement of outcomes	Low	Low	Moderate	Low	Moderate	Low
Selection of the reported result	Low	Low	Low	Low	Low	Low
Overall risk of bias	Low	Low	Moderate	Moderate	Moderate	Moderate

### 3.3 Risk-of-bias assessment of the included studies

Both the included RCTs (17, 20) reported using appropriate random sequence generation methods, resulting in a low risk of bias in this domain. However, none of the trials described specific allocation concealment procedures, and this aspect was categorized as having an “unclear risk” of bias. Due to the inherent limitations of anticoagulation interventions, neither trials implemented blinding of intervention providers or study participants, resulting in a high risk of bias in this domain. Furthermore, both the trials failed to explicitly state whether the outcome assessors were blinded, leading to an “unclear risk” of bias in this aspect.​ Regarding the completeness of outcome data, the trial conducted by Lee (20) was deemed as having a high risk of bias, whereas that conducted by Choi (17) demonstrated a low risk. Both studies comprehensively reported all prespecified primary outcomes, resulting in a low risk of selective reporting bias. The overall risk-of-bias assessment of the included studies is shown in Figure 1.

**Figure 1:**
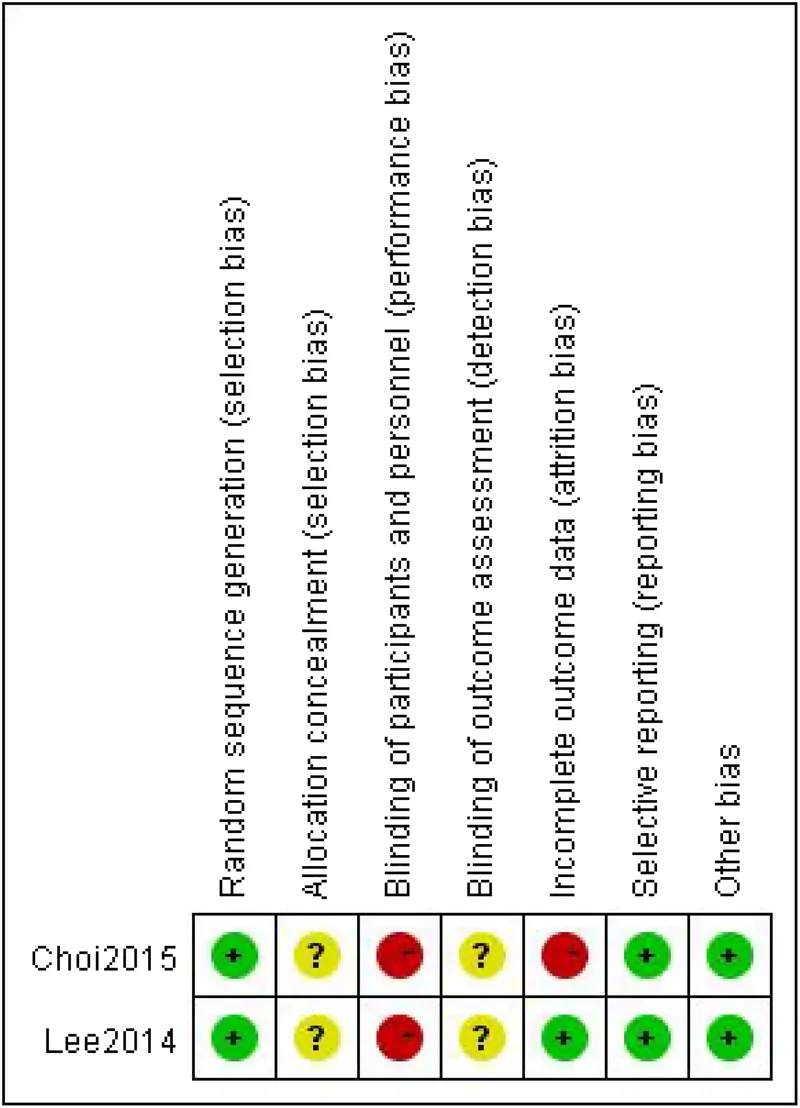
Summary of risks of bias.

### 3.4 Analysis of outcomes

#### 3.4.1 Filter lifespan

Five studies (18, 21–24) reported filter lifespan. Considerable heterogeneity was observed among the studies (*P* < 0.00001, I^2^ = 95%). The overall pooled MD in filter lifespan was 5.82 h (95% CI [4.74, 6.89]; *P* < 0.00001) (Figure 2). Subgroup analysis based on patient population showed an MD of 2.66 h (95% CI [1.38, 3.95]; I^2^ = 28%) in the ICU subgroup and 13.15 h (95% CI [11.19, 15.10]; I^2^ = 0%) in the high-bleeding-risk subgroup, with a statistically significant subgroup difference (*P* < 0.00001).

**Figure 2:**
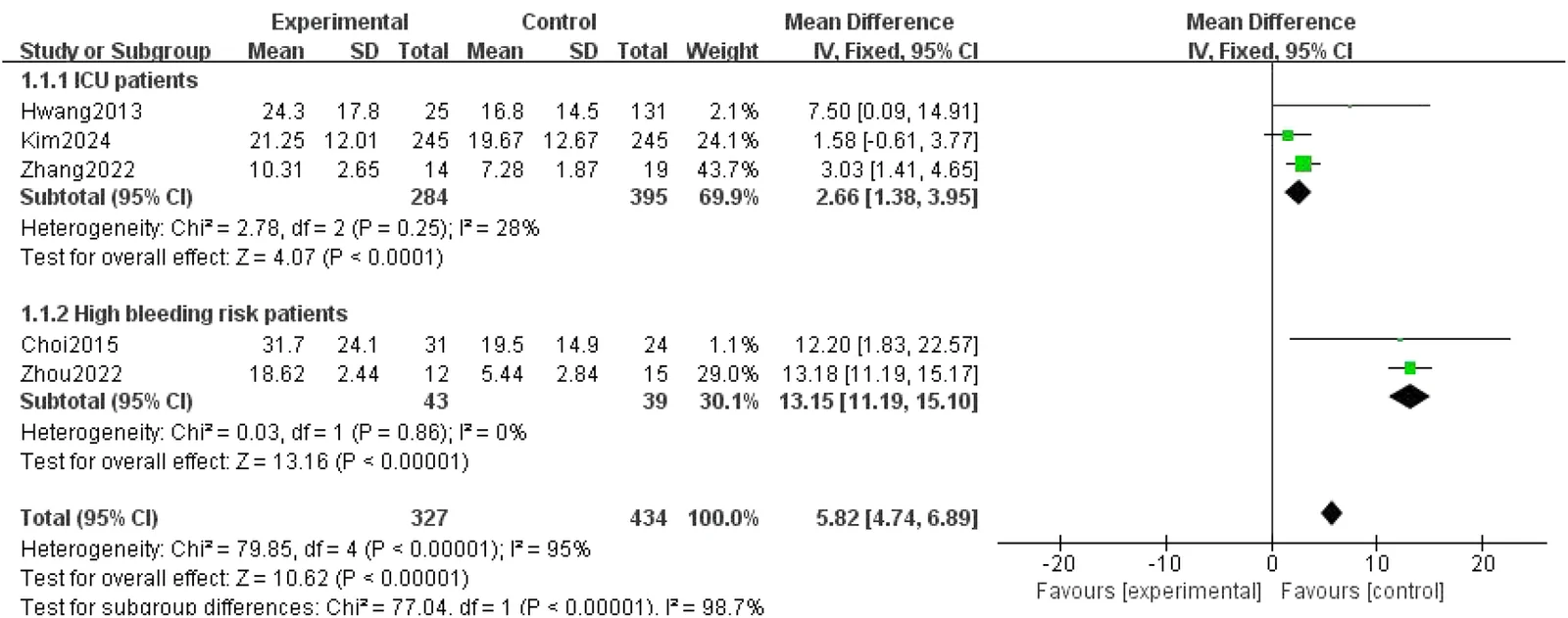
Forest plot comparing the filter lifespan between the nafamostat mesilate and no-anticoagulation group. CI, confidence interval; IV, intravenous; SD, standard deviation.

#### 3.4.2 Filter usage count

Three studies (17, 21, 24) reported filter usage count as an outcome measure. No heterogeneity was detected (*P* = 0.91, I^2^ = 0%), and a fixed-effects model showed a significantly lower count in the NM group (MD = -0.79; 95% CI [−0.97, −0.62]; *P* < 0.00001; Figure 3).

**Figure 3:**
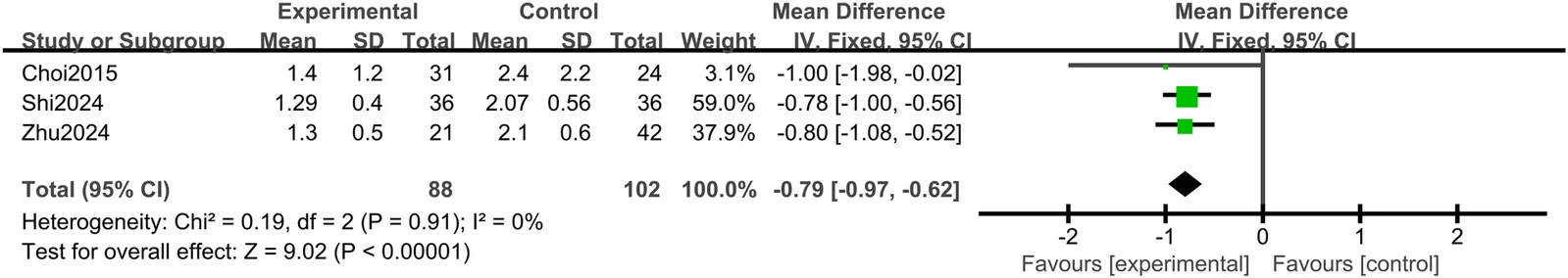
Forest plot comparing filter usage count​ between the nafamostat mesilate and no-anticoagulation group. CI, confidence interval; IV, intravenous; SD, standard deviation.

#### 3.4.3 Coagulation indicators

Five studies, three studies, and four studies reported APTT, PT, and INR as coagulation indicators, respectively. A random-effects model was used for APTT due to moderate heterogeneity (*P* = 0.07, I^2^ = 54%), while fixed-effects models were applied for PT (*P* = 0.75, I^2^ = 0%) and INR (*P* = 0.95, I^2^ = 0%). None of the indicators differed significantly between groups: APTT (MD = 0.23; 95% CI [−3.09, 3.55]; *P* = 0.89), PT (MD = 0.08; 95% CI [−0.90, 1.05]; *P* = 0.88), INR (MD = 0.02; 95% CI [−0.05, 0.09]; *P* = 0.60) (Figures 4a-c).

**Figure 4:**
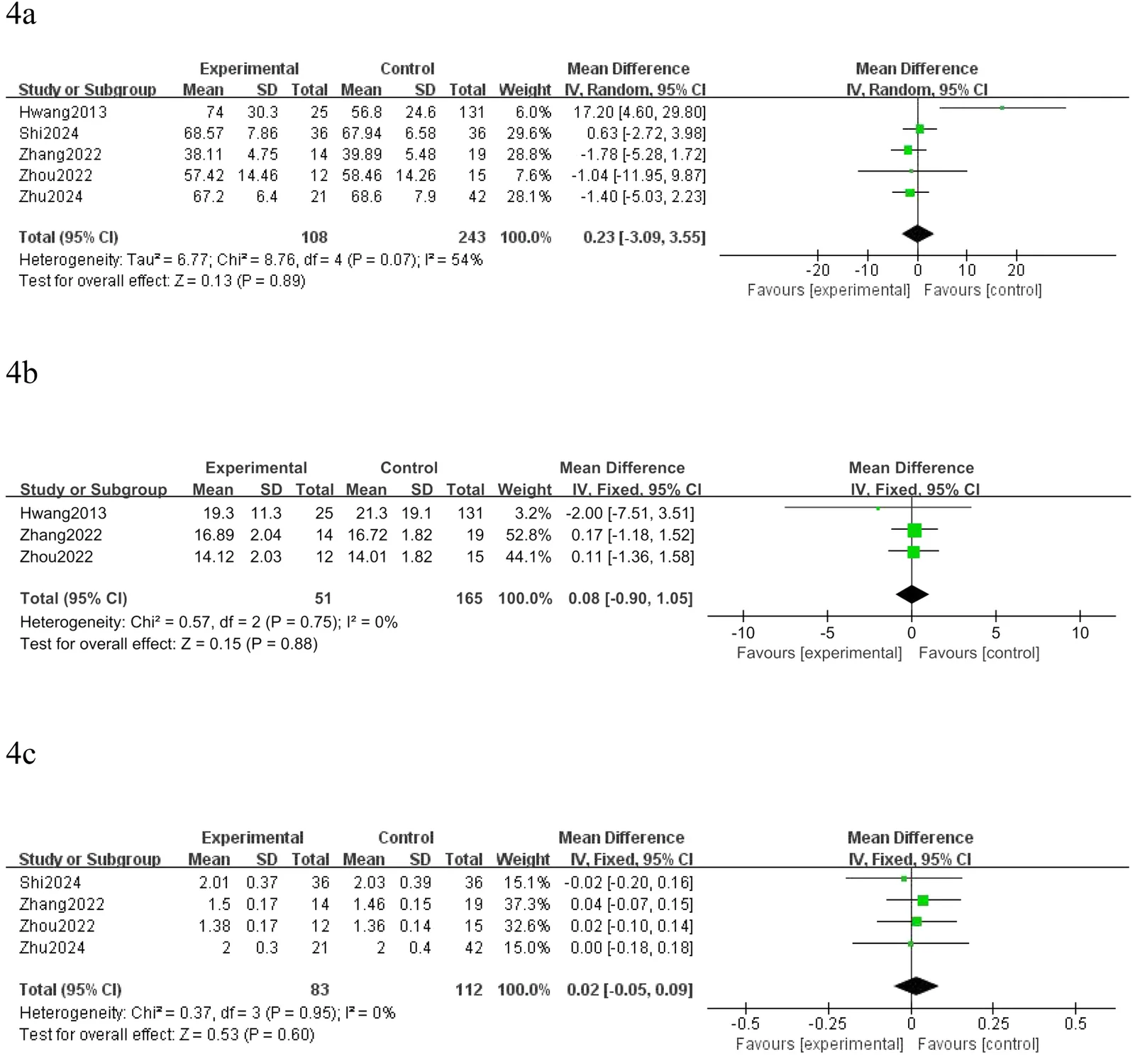
Forest plot comparing coagulation indicators between the nafamostat mesilate and no-anticoagulation group. **(a)** Forest plot of APTT. **(b)** Forest plot of PT. **(c)** Forest plot of INR. APTT, activated partial thromboplastin time; PT, prothrombin time; INR, international normalized ratio; CI, confidence interval; IV, intravenous; SD, standard deviation.

#### 3.4.4 Bleeding events

Five studies (18, 21–24) reported bleeding events as an outcome. No heterogeneity (*P* = 0.82, I^2^ = 0%) was found, and the fixed-effects model yielded no significant difference (OR=1.00; 95% CI [0.51, 1.96]; *P* = 0.99; Figure 5).

**Figure 5:**
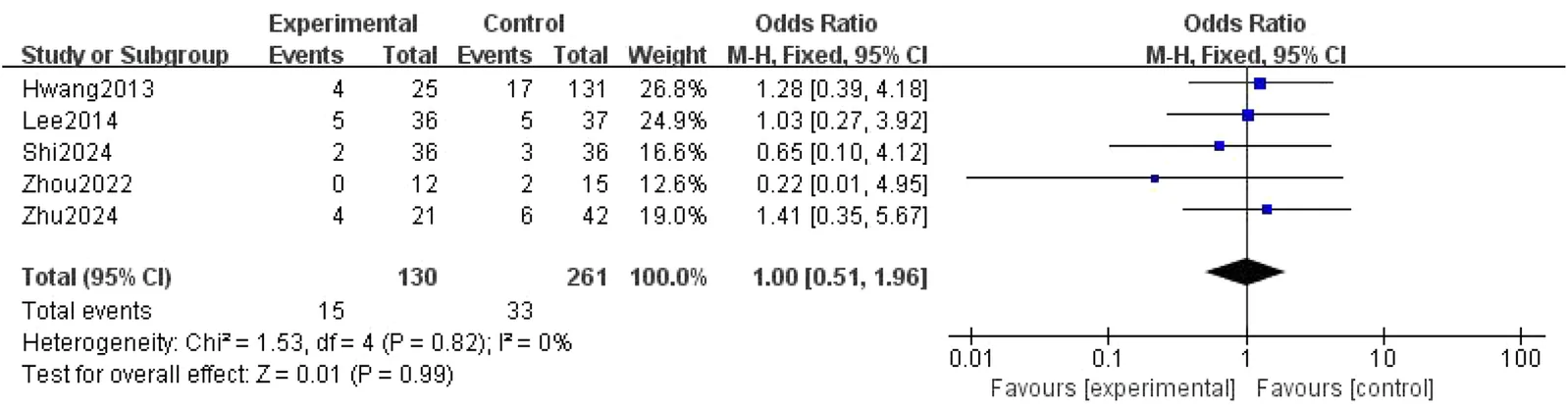
Forest plot comparing bleeding events between the nafamostat mesilate and no-anticoagulation group. CI, confidence interval; IV, intravenous; SD, standard deviation.

#### 3.4.5 In-hospital mortality

Four studies (18–20, 22) reported in-hospital mortality as an outcome. No significant heterogeneity (*P* = 0.23, I^2^ = 30%) was observed, and the fixed-effects model showed a significant reduction with NM (OR=0.68; 95% CI [0.50, 0.94]; *P* = 0.02; Figure 6).

**Figure 6:**
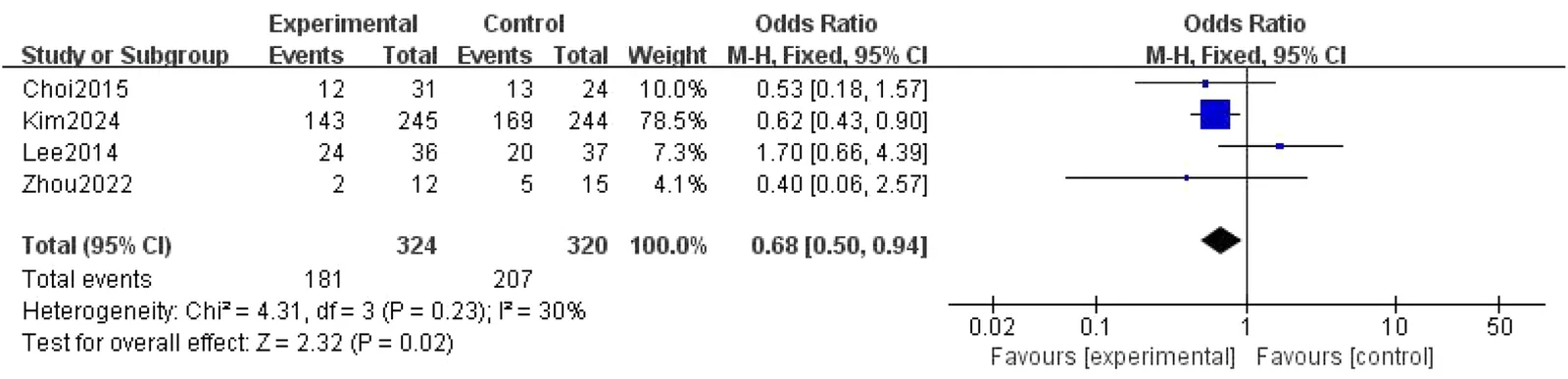
Forest plot comparing in-hospital mortality between the nafamostat mesilate and no-anticoagulation group. CI, confidence interval; IV, intravenous; SD, standard deviation.

#### 3.4.6 Sensitivity and publication bias

Sensitivity analysis was performed by sequentially removing individual studies. Since the availability of a sufficient number of studies, we conducted a sensitivity analysis on the filter lifespan, bleeding events, and in-hospital mortality. The overall pooled effect estimate for bleeding events remained stable, whereas the pooled effect estimates for filter lifespan and in-hospital mortality were no longer statistically significant after excluding the study by Kim et al. (19) (Supplementary Material 2). This loss of significance upon excluding Kim et al. (19) is attributable to its dominant weight and large sample size, which drove the overall estimates. Its removal left smaller studies with insufficient power, while high heterogeneity for filter lifespan further contributed to the instability. Egger's regression analysis showed that no significant publication bias was found in the main outcome of filter lifespan (*P* = 0.780).

### 3.5 Quality of evidence assessment (GRADE)

The certainty of the evidence was rated according to the GRADE method. The overall quality of the evidence ranged from low to moderate, indicating that further research may significantly alter the effect estimates. The summary of the results is presented in Supplementary material 3.

## 4 Discussion

This meta-analysis provides a focused systematic evaluation of the anticoagulant efficacy and safety of NM versus no-anticoagulation regimen in CRRT. Our results demonstrate that the NM regimen significantly prolongs filter lifespan and reduces the number of filters required per patient, without significantly altering systemic coagulation parameters (APTT, PT, INR) or increasing the risk of bleeding events. Regarding mortality, while a trend toward reduction was observed, this finding should be interpreted with caution as it was not robust in sensitivity analysis. These findings are consistent with a recent broader meta-analysis that reported similar benefits of nafamostat versus no-anticoagulation in blood purification modalities (25).

The prolonged filter lifespan with NM aligns with its pharmacological mechanism as a serine protease inhibitor, which blocks the intrinsic coagulation pathway by inhibiting factors XIIa, XIa, and kallikrein, thereby delaying fibrin deposition (26). In contrast, the no-anticoagulation regimen relies on saline flushes that transiently dilute coagulation factors but fail to inhibit the contact activation cascade (27). The significant heterogeneity (I^2^ = 95%) underscores the role of clinical variables, with subgroup analysis revealing maximal benefit in high-bleeding-risk patients, where no-anticoagulation is least effective. This pooled finding is consistent with previous external meta-analyses that demonstrated the superior efficacy of nafamostat in prolonging filter lifespan (11, 25), reinforcing the clinical and operational value of NM in this specific population. Furthermore, the prolonged filter lifespan with NM may yield operational benefits beyond clinical efficacy. However, these estimates are highly variable across settings and should be interpreted as highlighting potential efficiencies rather than definitive economic outcomes. Importantly, the effect is modulated by CRRT parameters such as AN69 membrane biocompatibility and blood flow rates (150–200 mL/min), which NM enhances pharmacologically. Therefore, while effective broadly, NM is strongly recommended as the anticoagulant of choice, particularly for patients with a high bleeding risk in whom its mechanistic advantage offers the greatest relative clinical and operational benefit.

The unchanged coagulation parameters can be attributed to the pharmacological properties of NM. Although NM forms effective anticoagulant concentrations within the extracorporeal circuit, it is rapidly hydrolyzed and inactivated by carboxylesterases upon entering the systemic circulation. Consequently, it is unlikely to cause significant alterations in systemic coagulation markers, such as APTT or PT (28). The short half-life of NM allows its clearance during CRRT, with its anticoagulant effect rapidly declining to undetectable levels after treatment cessation (29). This pharmacokinetic profile enables superior bleeding risk control, thereby minimizing systemic hemorrhage risk to the greatest extent. Zhan et al. demonstrated that NM undergoes rapid hydrolysis by plasma esterases after the blood exits the extracorporeal circuit, resulting in a significantly lower systemic bleeding risk, compared with heparin-based agents (*P* < 0.001) (30). This study further confirmed that even in high-bleeding-risk populations, there was no significant difference in the risk of bleeding events between the NM and NA groups (*P* = 0.53), providing evidence supporting the use of NM in high-bleeding-risk patients.​

Regarding mortality, the primary pooled analysis of four studies suggested a potential benefit for NM. However, this finding should be interpreted with considerable caution. A sensitivity analysis demonstrated that the statistical significance was entirely dependent on the inclusion of one large study. When this study was excluded, the effect was no longer significant. Therefore, while a trend favoring NM is noted, the current evidence is insufficient to confirm a mortality benefit. This instability highlights the limitation of the available data, which are derived from a small number of studies with varying designs and patient populations. The potential effect of NM on survival remains an open question that must be addressed in future, adequately powered randomized controlled trials.

Beyond the risk of bleeding, which was not increased with NM, it is important to consider other NM-specific adverse effects. The drug's profile includes potential risks such as hyperkalemia, anaphylaxis, and hypotension (12, 28). While our quantitative analysis was constrained by reporting limitations, the available data from included studies and broader literature suggest these events are infrequent but warrant vigilance. The localized action and short half-life of NM may contribute to its overall favorable safety profile compared to systemic anticoagulants. Nonetheless, future studies should prioritize standardized and comprehensive reporting of all adverse events to facilitate a more robust safety evaluation.

This meta-analysis confirms NM as an effective and safe alternative to no-anticoagulation in CRRT. Clinical decision-making often includes RCA, with choices influenced by non-pharmacologic factors, highlighting the need for direct NM-RCA comparative trials. Implementation beyond East Asia faces barriers (e.g., regulatory approval, supply chain, staff familiarity) but is aided by NM's simple administration and reduced monitoring needs. A feasibility-scalability framework indicates NM is initially viable in specialized centers, but broader adoption requires addressing systemic challenges.

This meta-analysis demonstrated significant heterogeneity among the included studies for some outcomes and filter lifespan (I^2^ = 95%), requiring a cautious interpretation of these results. The primary sources of heterogeneity include variations in CRRT modalities, pharmacodynamic variations arising from NM dosage fluctuations affecting anticoagulation intensity, and divergent baseline patient risk profiles—for instance, post-cardiovascular surgery patients exhibit significantly more severe coagulation disorders, compared with the general critically ill populations.​ It should be noted that the current evidence is derived exclusively from East Asian populations, namely, China and South Korea. Given that the metabolism of NM may be influenced by genetic polymorphisms, its applicability to European and American populations requires further validation.

This study has the following limitations. First, retrospective cohort studies were predominant among the included studies, potentially introducing confounding factors that may affect the effect estimates. Furthermore, CRRT circuit priming methods and saline flush frequency lacked standardization, potentially contributing to heterogeneity. Third, secondary outcomes were inconsistently reported; only Kim et al. documented length of stay in the ICU, precluding a cost-effectiveness analysis. Fourth, the generalizability of our findings is limited. All included studies were conducted in East Asia. Genetic and pharmacokinetic differences in NM metabolism may restrict the direct applicability of our results to non-East Asian populations. Consequently, the observed efficacy and safety of NM require validation in Western populations through future multinational trials. Fifth, detailed information on dialysis membrane types and CRRT modalities was inconsistently reported across studies, precluding subgroup analyses based on these technical parameters.

## 5 Conclusions

This study confirms that the NM anticoagulation regimen extends filter lifespan and reduces filter replacement frequency without increasing the risk of bleeding events. Regarding mortality, while a positive trend was observed, the pooled estimate was not robust and should be interpreted with caution. NM represents a viable alternative to anticoagulant-free protocols for critically ill patients undergoing CRRT, especially for those with a high bleeding risk. Future large-scale, multicenter RCTs with standardized technical parameters are warranted to validate its effect on mortality and to evaluate its efficacy in non-East Asian populations and pediatric patients.

## Data Availability

The original contributions presented in the study are included in the article/Supplementary Material, further inquiries can be directed to the corresponding author.
