# Supplementary material for: Meta-analysis of the effect of nafamostat mesilate versus no-anticoagulation regimens in adult patients undergoing continuous renal replacement therapy
Source: Front Med (Lausanne). 2026 Oct 21;13:1984576. doi: 10.3389/fmed.2026.1984576 (PMC13731358; doi:10.3389/fmed.2026.1984576)
Supplement: Supplementary file 1 [file Supplementaryfile1.docx]

Records identified through databases search (n=307):

Pubmed:65, CINAHL:21, MEDLINE: 49, Cochrane Library: 7, Web of Science: 22, CNKI: 49, Wanfang: 71, SinoMed: 23

Duplicate removed (n=112)

**Identification**

Records excluded (n=163):

irrelevant topic (n=85)

irrelevant population (n=32)

case reports (n=19)

meta-analysis or systematic review (n=27)

Records screened by tittle and abstract after duplicates removed (n=195)

Reports sought for retrieval

(n=32)

Reports not retrieved

(n=0)

**Screening**

Reports excluded(n=24):

inappropriate intervention (n=18) not reporting outcomes of interest (n=6)

Reports assessed for eligibility

(n=32)

Studies included in meta-analysis(n=8)

**Included**

Supplementary Figure 1. PRISMA flowchart.
