# Supplementary material for: Meta-analysis of the effect of nafamostat mesilate versus no-anticoagulation regimens in adult patients undergoing continuous renal replacement therapy
Source: Front Med (Lausanne). 2026 Oct 21;13:1984576. doi: 10.3389/fmed.2026.1984576 (PMC13731358; doi:10.3389/fmed.2026.1984576)
Supplement: Supplementary file 2 [file Supplementaryfile2.docx]

A

B

C

Supplementary material 2. Sensitivity analysis on results stability regarding filter lifespan, bleeding events, and in-hospital mortality

(A) filter lifespan, (B) bleeding events, (C) in-hospital mortality
