# Supplementary material for: Meta-analysis of the effect of nafamostat mesilate versus no-anticoagulation regimens in adult patients undergoing continuous renal replacement therapy
Source: Front Med (Lausanne). 2026 Oct 21;13:1984576. doi: 10.3389/fmed.2026.1984576 (PMC13731358; doi:10.3389/fmed.2026.1984576)
Supplement: Supplementary file 3 [file Supplementaryfile3.docx]

Supplementary material 3. Quality of evidence (GRADE)

| Outcome | Risk of Bias | Inconsistency | Indirectness | Imprecision | Publication bias | Quality of  evidence |
| --- | --- | --- | --- | --- | --- | --- |
| Filter Lifespan | Serious | Serious | Not Serious | Not Serious | Undetected | ⊕⊕⊖⊖;low ^a,b^ |
| Filter Usage Count | Serious | Not Serious | Not Serious | Serious | Undetected | ⊕⊕⊖⊖; low ^a,c^ |
| Coagulation Indicators | Serious | Not Serious | Not Serious | Serious | Undetected | ⊕⊕⊖⊖; low ^a,c^ |
| Bleeding Events | Serious | Not Serious | Not Serious | Not Serious | Undetected | ⊕⊕⊕⊖; Moderate ^a^ |
| In-hospital Mortality | Serious | Not Serious | Not Serious | Not Serious | Undetected | ⊕⊕⊕⊖; Moderate ^a^ |

Note: ^a^ Downgraded one level due to high risk of bias (incomplete blinding). ^b^ Downgraded one level due to large heterogeneity between studies. ^c^ Downgraded one level due to imprecision (defined as wide confidence intervals including no effect and/or low overall sample size).
